# Supplementary material for: A sweet new set of inducible and constitutive promoters in Haloferax volcanii
Source: Front Microbiol. 2023 Aug 10;14:1204876. doi: 10.3389/fmicb.2023.1204876 (PMC10448506; doi:10.3389/fmicb.2023.1204876)
Supplement: Supplementary file 1 [file Table_1.DOCX]

Table S1. *Haloferax volcanii*-specific ribodepletion probes.

| Sequence |
| --- |
| CCTACCCCGGACGTACCCCTCGAGCCTTCCGGTCCCAAGCCACGTGGTTT |
| ATCGTCATCACTGTAGTCGGAGCTGGTGAGATGTCCGGCGTTGAGTCCAA |
| GTTCGTTTTTCAAAACGTACGACGTAACATCGGCTTCACTCGAGTCATAC |
| GACCTACCGTTGCCCGTTCCTTCCTCCGCTTTAGCAGCGGCAGTCCTTCT |
| GCACCGCCCTGTTCGGGGTGCTTTTCAGCGTTCGCTCACGCTACTTGTTC |
| TACGACGTTCGTAAGTTCGGAGTTTGACAGGGAAGCGAACTCCTCTCGGA |
| CCTACTAAGATGTTTCAATTCGGAACGTTCCCCATTGCGCGAGGCAATTG |
| CTACGGAATCGTGTCATAAGACACGCCGCCAGGTCTCGGAGGTGGATTTG |
| ACCACGTCTCAGCGACAGCCTGCAGTAAAGCTCTATAGGGTCTTCGCTTC |
| AGGAGGTGATCCAGCCGCAGATTCCCCTACGGCTACCTTGTTACGACTTA |
| TCGTTTACAGCTAGGACTACCCGGGTATCTAATCCGGTTCGAGACCCTAG |
| GTTTCGGGTCGCATCCGGTTGGCTCCCCGCCCTTGAAGACGGTGGCCCTG |
| CCCGCTCGGTGTTTCCTAAAGCGTCGACGTTTGGTGCAAGAAGGTACTGG |
| GTAGCAAGCCACCGGGTCGATATGTGCTCTTGCCGGTGACGACTCTGTTA |
| CTGCCTCGGTGGCCCGCTGGCGCGGGTACCTGTGTAATGGCTCCTACCTA |
| TTCTGTCATCTACGGGTCCCATGAATGAACCTCGTAGGTTCGCTAGACCA |
| GTGCAAGGAGCAGGGACGTATTCACCGCACTCTATTGAAATGCGATTACT |
| AGTCTATCGAACTCGTCTTCTACGAGTGTCCTCAGTGGAACTTCTTTTCC |
| CACCGTTCGTTCGCTTCGTGCCGTTACGGCTTCCACGAATTTCGGTGATT |
| GCCCGGCTACCGGTGTCCTCCGCCAGGAGTGAGAGTCGCAGTCACTAACG |
| GATCTCGGTACGAGCTTCTTGCTTGTCTTTTCACGGGCTCTAGGTACCGC |
| GTCGGATCCGTCTTCTCGAGGTGCTTTCGCCATCGGTGGTCCGTCCAGGA |
| GCTGTTGCCAAGTTCGATGGGCCTTTCACCCCTACACCAGAGTCACGAGA |
| TCGAAACTGCTGCTGGCAATTAGAAGTGCGGGTCTCGCTCGTTGCCTGAC |
| AATAGGCGAACAACCTCACCCTTGCCTGCTTCTGCACAGGCAGGATGGAG |
| CGTATCGGTTTCCCTATGCCTCCGGGGGTTCACCCCTTAGACTCGCCAGT |
| AGACTCCGCACTGGAACGTACTGTTCACCGGGCCCAACGTTGGGACAGTG |
| GAATGTGGCTTGGACTGTTAGTGCTCGCGGGCTTAACAACTCGTTACCTC |
| CAGTTGAGCTGGCGGATTTCCCGATGAACCTTCGTGGCCGGCTACGGACG |
| ACCTGGCTCTGGGTTGTCTCCCTCACGGTGCACAAGCTTACCCCGCACAC |
| TTCCTCGACCGCCTTACGGTCGAGAAAAGCGAGGACTATATGCCCTCGCA |
| AACTAGCATGGCTAAATCGGACCCCAATAGCAATGACCTCCGGCAGGATC |
| GATTTTCGTTGCTGCACGGTCCACACGAGTTCTCACCCGTGCTTCCACCC |
| CCGCCGTTGACGGGTCCTTCGTCCCATTGTAATGGGTGTTCAGATACCCG |
| AGTGACCGTACGAGTCCTTGCGGATTTGCGGTCACCTATGTTGTTACTAG |
| ACACCCCCAATAGATAGCAGCCGACCTGTCTCACGACGGTCTAAACCCAG |
| ATGAGGGCGAGTTTCAGCCCTCAATCCGAACTACGATCGAGTTTAGGAGA |
| CGGTCGCTCTACCTCACGAACTATCTCATCAGAGGTCATGCTTCGACATG |
| CGACGCCTCGAACCTCGGCCGGTAAGCTGTTACGCTATTCTTAGAGGGTA |
| TTAGATGCGTTCAGCTCTTACCCCGTGGTGCGTAGCTGCCCGGCATCTGC |
| ACCTCCCGGCTGTTTAGGGCTCGAGACAATCTTCAATCACACTTAATCCA |
| ACGGTACGAGCTGACGGCGGCCATGCACCTCCTCTCTACAGCGTCGTGAT |
| GAGTGTTTAGTCTTCGCAGTTGATGCCTGCGAAATTCACGAGGGATATCC |
| GAGTCACTGCGACCTGCTCGGTCAAGAGCAGGCATCCCTTATCGCGAACT |
| AGTTCTTTCGTCGAGTGACCTTGTATGCCACGTCGCTCTATCGCCAACTG |
| CATTCATGCAAGCCGCTACTGAAGCGGCAAGGTACTACGCTACCTTAAGA |
| GAGATAGCAACCCATTGTCTCGACCATTGTAGCCCGCGTGTAGCCCAGCT |
| CTCGCGTGAGGAAATCTTTGGGCGCGCTCGATATCTTTTCGAGCGCGTAC |
| GTTTGGACTGTGTCGCGTTTACTCGCCGTTACTAACGACATCGCGGATTG |
| TATCGGTCATCACTTGAGCTGCCGGTATTACCGCGGCGGCTGGCACCGGT |
| CCAACTCTAACGGGCCTCCACGCAGCTTTCGCCACGCTTCGCCCTGCTCC |
| CCGAGGAACCCTTGCGCGTAAGGCCGTCGGGGTTCACACCCGACTCTCGC |
| CTCCGGTTGTAGTGCTCCCCCGCCAATTCCTTTAAGTTTCATCCTTGCGG |
| GTACACCAGTGGCACCCATTCGTAGTTCCTCTCGTACTATACGAACGTTC |
| TCGGAGATCCTCAGTTCTTTCCCTCCATGCGGGTCCCTGAGGCTTATCGC |
| GAGACTTCACATAAGACCCAGGTCCGTTATGGGTAGTCCGTACACCACAT |
| TCATCGGCTCTCGAGCCGAGCTATCCACCAGCTGGCACAGTAGCCAGTTG |
| CCCACCGTCTACCTAATCGGCCGCAGCCACATCCTACAGCGCCTGAGCGT |
| AGTTTGGCCACGTGTTACTGAGCTTTTCGCCACGAGTATGAACTCGTGCA |
| CCGAATTCCCTAACGTCGGTTGATCCCGACAGGCCTTGGCTTGCTCTGCC |
| CCCTTTCATCTAATTCGAATTACGGTTAGACTTAGGATCGACTAACCCTC |
| TTCCAGCATGACTCCCGTATGAACTATTAGCCTCAGTTTCCCGAGGTTAT |
| GGAACAACCCCGGGATCTGGTTCTTATGTTACGGGGTTTTCACCCTGTAT |
| GGCTCGCTTCTCGGCTTCCCTACGGCACAACACAGGCTCGTAGCCTGTGT |
| CAGATTCCGTCTCCGGGCTCTTGCTCTCACAACCCGTACCGATTATCGGC |
| GACACTCGTTGGGAATGTCACTGTCAGACTTCCGTTTGCTCTTGCGCTCT |
| TCGCACTTGCGTGCAGTGTAAAGGTTTCGCGCCTGCTGCGCCCCGTAGGG |
| CCCAGATTCGACCACCGTGTGGTGGCCTCATCCGGACCTCACTCGGGTGC |
